# Comparative Effects of Biodynes, Tocotrienol-Rich Fraction, and Tocopherol in Enhancing Collagen Synthesis and Inhibiting Collagen Degradation in Stress-Induced Premature Senescence Model of Human Diploid Fibroblasts

**DOI:** 10.1155/2013/298574

**Published:** 2013-12-14

**Authors:** Suzana Makpol, Faidruz Azura Jam, Shy Cian Khor, Zahariah Ismail, Yasmin Anum Mohd Yusof, Wan Zurinah Wan Ngah

**Affiliations:** ^1^Department of Biochemistry, Faculty of Medicine, Universiti Kebangsaan Malaysia, Jalan Raja Muda Abdul Aziz, 50300 Kuala Lumpur, Malaysia; ^2^R&D Plantation and Agri-Business Division, Sime Darby Research Sdn Bhd, 42960 Carey Island, Selangor, Malaysia

## Abstract

Biodynes, tocotrienol-rich fraction (TRF), and tocopherol have shown antiaging properties. However, the combined effects of these compounds on skin aging are yet to be investigated. This study aimed to elucidate the skin aging effects of biodynes, TRF, and tocopherol on stress-induced premature senescence (SIPS) model of human diploid fibroblasts (HDFs) by determining the expression of collagen and MMPs at gene and protein levels. Primary HDFs were treated with biodynes, TRF, and tocopherol prior to hydrogen peroxide (H_2_O_2_) exposure. The expression of *COL1A1, COL3A1, MMP1, MMP2, MMP3,* and *MMP9* genes was determined by qRT-PCR. Type I and type III procollagen proteins were measured by Western blotting while the activities of MMPs were quantified by fluorometric Sensolyte MMP Kit. Our results showed that biodynes, TRF, and tocopherol upregulated collagen genes and downregulated *MMP* genes (*P* < 0.05). Type I procollagen and type III procollagen protein levels were significantly increased in response to biodynes, TRF, and tocopherol treatment (*P* < 0.05) with reduction in MMP-1, MMP-2, MMP-3, and MMP-9 activities (*P* < 0.05). These findings indicated that biodynes, TRF, and tocopherol effectively enhanced collagen synthesis and inhibited collagen degradation and therefore may protect the skin from aging.

## 1. Introduction

Human skin which consists of epidermis, dermis, and subcutaneous tissues provides a shielding layer for internal organs. During chronological aging, increased wrinkling, sagging, pigmentation, fragility, and lack of moisture plus elasticity are the universal manifestations observed on the skin. Skin aging can be intrinsic, which is genetically determined and extrinsic, which is caused by environmental exposure such as UV light. Oxidative stress is one of the factors that contribute to skin aging [1, 2].

Fibroblasts which are the crucial collagen-producing cells provide flatten appearance and elasticity to the skin in cooperation with collagen. However, fibroblasts have collapsed appearance with little cytoplasm when they aged [3, 4]. Therefore evaluating the loss of collagen, either decreased synthesis or increased degradation, is important in analyzing the factors that may contribute to skin aging [5]. Matrix metalloproteinases (MMPs) play an important role in regulating the turnover of collagen. In aged skin, the elevated level of MMPs caused increased collagen degradation and deterioration of skin structure [6]. Previous study which used stress induced premature senescence (SIPS) model of human diploid fibroblasts has shown the role of MMPs in regulating collagen degradation [7, 8].

Collagen fibers comprised approximately 75% of the dry weight of the dermis [9]. Total collagen in skin will decrease with age. Previous study showed that collagen markers such as type I C-terminal propeptide (PICP) did not show any detectable increase during adolescence but decreased towards adult concentrations after the age of puberty while cross-linked C-terminal telopeptide of type I collagen (ICTP) and procollagen type III N-terminal propeptide (p3NP) increased in pubertal-aged children before decreasing towards adults concentrations [10]. Aged individuals have been reported to have lower collagen levels in skin as compared to young individuals while the amount of elastic materials and associated fibro-hexis or fiber breakdown can be large and is probably responsible for wrinkle formation seen in photoaged skin [2, 8, 11, 12].

MMPs are a family of zinc containing proteases with various substrate specificities, cellular sources, and inducers [4]. They degrade the stable components in extracellular matrix (ECM) such as collagens, gelatin, elastin, laminin, and basement membranes. MMPs levels in skin increase with age [6]. It has been suggested that the presence of damaged collagen may act in some manner to downregulate collagen synthesis. Study has shown that damage to type I collagen in three-dimensional *in vitro* culture following MMP-1 treatment has similar ultrastructural appearance to the damage seen *in vivo* in aged skin [13].

Development of aging is associated with oxidative stress as postulated in the free radical theory of aging [14]. Free radicals such as reactive oxygen species (ROS), which can be produced intrinsically through normal metabolic processes or from exogenous agents, attack cellular structures like DNA and protein causing to continuous accumulation of cellular damage. *In vitro*, the oxidative stress condition can be manipulated in order to study the aging process. For instance, studies have shown that exposure to ultraviolet or hydrogen peroxide (H_2_O_2_) was able to elevate ROS content and induce premature senescence in young fibroblasts [15, 16]. H_2_O_2_ is the oxidant species that can induce oxidative damage in human fibroblasts and produce similar characteristics as senescent cells [17].

Since oxidative stress is vital in aging, compounds with antioxidant properties might be beneficial in preventing aging. In this study we evaluated the combined effects of biodynes, tocotrienol-rich fraction (TRF), and tocopherol in promoting skin regeneration prior to oxidative stress exposure. TRF is comprised of all forms of tocotrienols and *α*-tocopherol. Tocopherol and tocotrienol are isomers of vitamin E. The difference in their chemical structure contributes to the different efficacy and potential as antioxidant [18]. Biodynes is an active compound which is derived from *Saccharomyces cerevisiae*. Recent studies showed that it acts as an antiaging compound due to its collagen synthesis promoting effect. When combined with other active compounds, it may work synergistically [19].

In this study we aimed to elucidate the molecular mechanism of biodynes, tocotrienol-rich fraction, and tocopherol in preventing skin aging. We would like to determine whether single treatment is giving thrilling outcomes or the synergic effects of combined compounds provide a better impact in preventing skin aging by determining collagen biosynthesis and degradation in HDFs.

## 2. Materials and Methods

### 2.1. Cell Culture and Treatment Protocols

This research has been approved by Ethics Committee of Universiti Kebangsaan Malaysia (Approval Project Code: FF-328-2009). Primary human diploid fibroblasts (HDFs) were derived from the circumcision foreskins of three young male subjects, aged between 9 and 12 years old. Written informed consents were obtained from parents of all subjects. The samples were aseptically collected and washed several times with 75% alcohol and phosphate buffered saline (PBS) containing 1% antibiotic-antimycotic solution (PAA, Austria). After removing the epidermis, the pure dermis was cut into small pieces and transferred into a falcon tube containing 0.03% collagenase type I solution (Worthington Biochemical Corporation, USA). Pure dermis was digested in the incubator shaker at 37°C for 6 to 12 h. With PBS, the derived HDFs were washed and maintained in Dulbecco Modified Eagle Medium (DMEM) containing 10% fetal bovine serum (FBS) (PAA, Austria) and 1% antibiotic-antimycotic solution at 37°C in 5% CO_2_ humidified incubator. After 5 or 6 days, the HDFs were trypsinized and further expanded with expansion degree of 1 : 4 into a new T25 culture flask (Nunc, Denmark). When cell confluency reached 80 to 90%, serial passaging was performed until passage 4. The population doublings (PDs) were monitored throughout the experiment. For subsequent experiments, HDFs were treated with biodynes, TRF, or tocopherol and combination of biodynes, TRF, and tocopherol (BTT) at passage 4. After that, the cells were exposed to 20 *μ*M H_2_O_2_ for two weeks (prolong exposure to low concentration of H_2_O_2_) at passage 6. Immediately after two weeks of H_2_O_2_ exposure, the cells were harvested for further analysis.

Biodynes TRF (Arch Chemicals Inc. NJ, USA), tocotrienol-rich fraction (TRF) (Sime Darby Plantation Sdn. Bhd, Malaysia), and Copherol F 1300 C (Cognis Care Chemicals) were used to treat the HDFs prior to H_2_O_2_ exposure at a concentration of 1% (v/v), 500 *μ*g/mL, and 100 *μ*g/mL, respectively, either single or in combination.

### 2.2. Total RNA Extraction

Total RNA of HDFs in different groups was extracted by using TRI Reagent (Molecular Research Center, USA), according to manufacturer's instructions. Polyacrylcarrier (Molecular Research Center, USA) was added in each extraction to precipitate the total RNA. Extracted total RNA pellet was then washed with 75% ethanol and air-dried before being dissolved in RNase DNase free distilled water (Gibco, USA). Total RNA was stored at −80°C immediately after extraction. The yield and purity of the extracted total RNA were determined by using Nanodrop ND-1000 (Thermo Fisher Scientific, USA).

### 2.3. Primer Design and qRT-PCR

All primers were designed by using Primer 3 software (http://frodo.wi.mit.edu/primer3), with reference of Genbank (http://www.ncbi.nlm.nih.gov) database. The targets amplified by the primer pairs were characterized by BLAST (Basic Local Alignment Search Tool; http://blast.ncbi.nlm.nih.gov).  Table 1 shows the primers sequence for targeted genes in this study. Specificity of all primers was determined by using iScript One-Step RT-PCR Kit with SYBR Green (Biorad, USA). The size of the PCR products was then checked by running on 1.8% agarose gel prestained with ethidium bromide along with a 100 bp DNA step ladder (Promega, USA). Optimization of the qRT-PCR procedures was established by performing the standard curve. Four serial dilutions of total RNA were used: 0, 2, 4, 8 and 16. By using Bio-Rad iCycler and programmed protocol, each primer pair was optimized and the expression of all targeted genes was determined. The amplification protocol was as follows: cDNA synthesis at 50°C for 30 min, iScript reverse transcriptase inactivation at 94°C for 2 min, followed by 38 amplification cycles of denaturation at 94°C for 30 sec and 60°C (primer annealing and extension) for 30 sec. After the end of the last cycle, the melting curve was generated at 95°C for 1 min, 55°C for 1 min, and 60°C for 10 sec (70 cycles, increase set point temperature after cycle 2 by 0.5°C). Glyceraldehyde 3-phosphate dehydrogenase (*GAPDH*) was used as reference gene that acts as an internal reference to normalize the mRNA expression [20].

### 2.4. Western Blotting

To determine the amount of type I procollagen and type III procollagen protein, cells were lysated by using lysis buffer which was prepared by mixing complete protease inhibitor cocktail tablet (Roche, German) and RIPA buffer (Sigma-Aldrich, USA). Approximately 30 *μ*g of cell lysate was heated at 70°C for 10 min in the sample buffer. The samples were then separated on 4–12% bis-tris gel (Invitrogen, USA) by gel electrophoresis. After that, proteins were transferred onto nitrocellulose membrane and incubation of primary antibody was performed. Two primary antibodies were used which were anti-mouse monoclonal type I procollagen (Santa Cruz, USA) at 1 : 500 dilution and type III procollagen (Santa Cruz, USA) at 1 : 200 dilution. After incubation of secondary antibody, the proteins expression was detected by gel documentation and analysis MultiImage Light Cabinet 504551 (Alpha Innotech, USA). The band intensities were measured by using Image Master Total Lab software (Amersham Bioscience, Buckinghamshire, UK).

### 2.5. Determination of MMP-1, MMP-2, MMP-3, and MMP-9 Activities

The activity of MMP-1, 2, 3, and 9 was quantified by using the fluorometric SensoLyte 520 MMP assay kit (Anaspec, USA) according to the manufacturer's instructions. Briefly, cells were treated for 24 h. The supernatant of conditioned medium was collected and centrifuged for 15 min at 4°C, 10,000 g. Samples containing MMP were then incubated with 4-aminophenylmercuric acetate (APMA) to activate pro-MMP followed by initiating the enzyme reaction. The activity of MMP was detected by fluorescence microplate reader (Bio-Tek Instruments, USA) at excitation/emission wavelengths of 360 nm/460 nm.

### 2.6. Statistical Analysis

Experiments were performed in triplicates and data was analyzed by one-way analysis of variance (ANOVA) using SPSS statistic software. Significance was accepted at *P* < 0.05.

## 3. Results

### 3.1. Effect of Biodynes, Tocotrienol-Rich Fraction, and Tocopherol on Collagen Synthesis

**Figure 1:**
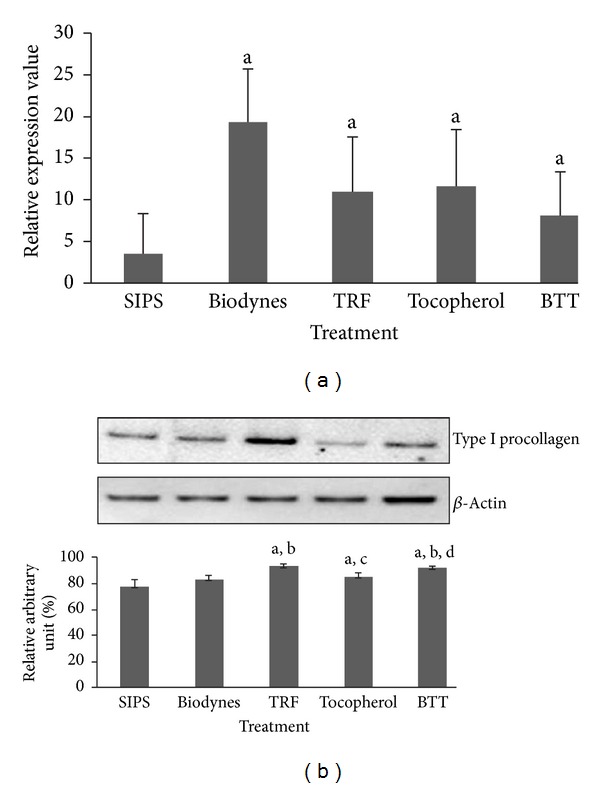
Effects of biodynes, TRF, and tocopherol on *COL1A1 *gene and procollagen type I protein expression in HDFs. *COL1A1 *expression (a). Biodynes, TRF, tocopherol, and BTT significantly increased *COL1A1* expression compared to SIPS. Procollagen type I protein expression (b). TRF, tocopherol, and BTT significantly increased the expression of procollagen type I. ^a^Denotes *P* < 0.05 compared to SIPS, ^b^denotes *P* < 0.05 compared to biodynes, ^c^denotes *P* < 0.05 compared to TRF, and ^d^denotes *P* < 0.05 compared to tocopherol. Data are presented as the mean of three experiments ±S.D, *n* = 6.

Biodynes, TRF, tocopherol and combined biodynes, TRF, and tocopherol (BTT) significantly increased the expression of *COL1A1* gene as compared to SIPS at 5.07-fold, 2.92-fold, 3.10-fold, and 2.13-fold, respectively (Figure 1(a)) (*P* < 0.05). However, the significant elevation was only found in HDFs treated with TRF, tocopherol, and BTT (*P* < 0.05) but not in biodynes-treated cells when the levels of type I procollagen protein were analyzed (Figure 1(b)).

**Figure 2:**
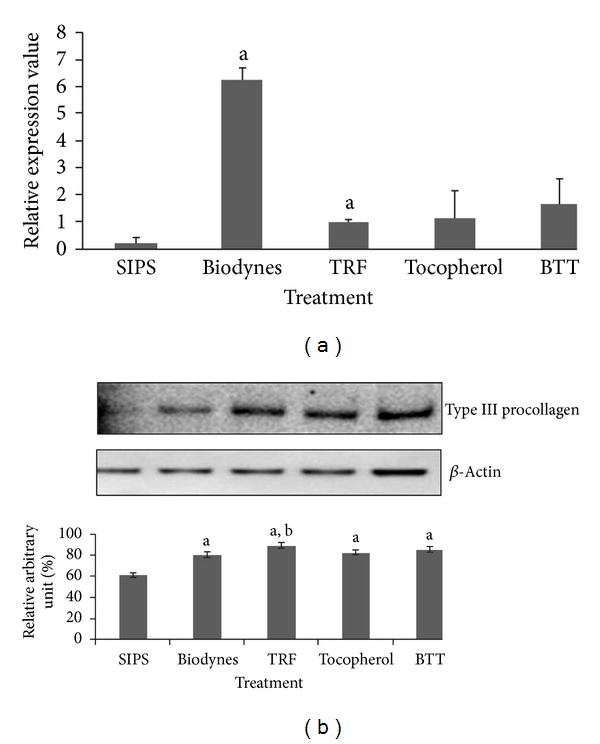
Effects of biodynes, TRF, and tocopherol on *COL3A1 *gene and procollagen type III protein expression in HDFs. *COL3A1 *expression (a). Biodynes and TRF significantly increased *COL3A1* expression compared to SIPS. Procollagen type III protein expression (b). Biodynes, TRF, tocopherol, and BTT significantly increased the expression of procollagen type III. ^a^Denotes *P* < 0.05 compared to SIPS, ^b^denotes *P* < 0.05 compared to biodynes. Data are presented as the mean of three experiments ±S.D, *n* = 6.

For *COL3A1 *expression, a significant upregulation by 9.63 and 1.22 fold over SIPS was observed in cells treated with biodynes and TRF respectively (Figure 2(a)) (*P* < 0.05). Expression of type III procollagen was significantly increased in all treated cells as compared to SIPS (Figure 2(b)) (*P* < 0.05).

### 3.2. Effect of Biodynes, Tocotrienol-Rich Fraction, and Tocopherol on Collagen Degradation

**Figure 3:**
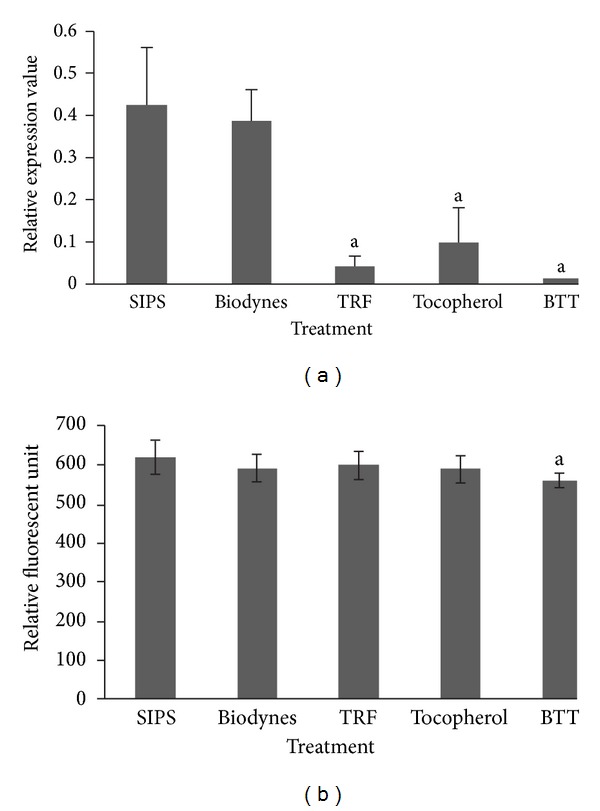
Effects of biodynes, TRF, and tocopherol on *MMP1 *gene expression and MMP-1 activity in HDFs. *MMP1 *expression (a). TRF, tocopherol, and BTT significantly downregulated *MMP1 *compared to SIPS. MMP-1 activity (b). BTT decreased the MMP-1 activity compared to SIPS. ^a^Denotes *P* < 0.05 compared to SIPS. Data are presented as the mean of three experiments ±S.D, *n* = 6.

**Figure 4:**
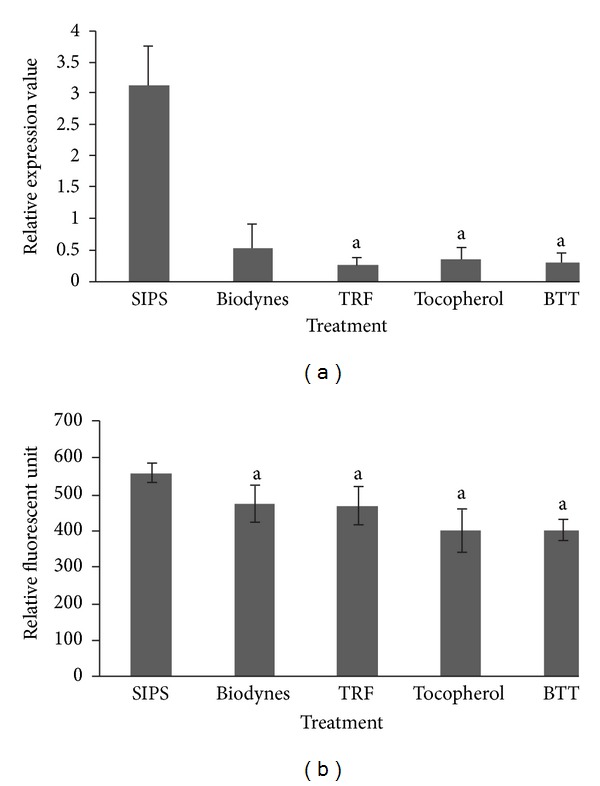
Effects of biodynes, TRF, and tocopherol on *MMP2 *gene expression and MMP-2 activity in HDFs. *MMP2 *expression (a). TRF, tocopherol and BTT downregulated *MMP2 *compared to SIPS. MMP-2 activity (b). Biodynes, TRF, tocopherol, and BTT significantly decreased, the MMP-2 activity compared to SIPS. ^a^Denotes *P* < 0.05 compared to SIPS. Data are presented as the mean of three experiments ±S.D, *n* = 6.

Treatment with TRF, tocopherol and BTT significantly down regulated *MMP1* gene as compared to SIPS at 0.10-fold, 0.23-fold and 0.03-fold respectively (Figure 3(a)) (*P* < 0.05). Analysis on MMP activities showed that only BTT significantly reduced MMP-1 activity (Figure 3(b)). For *MMP2 *gene expression, the result was similar to the expression of *MMPI *whereby TRF, tocopherol and BTT significantly decreased *MMP2* gene expression by 0.09-fold, 0.12, and 0.10, respectively, but not in biodynes-treated cells (Figure 4(a)). However, all treatment groups showed significant reduction in MMP-2 activity (Figure 4(b)) (*P* < 0.05).

**Figure 5:**
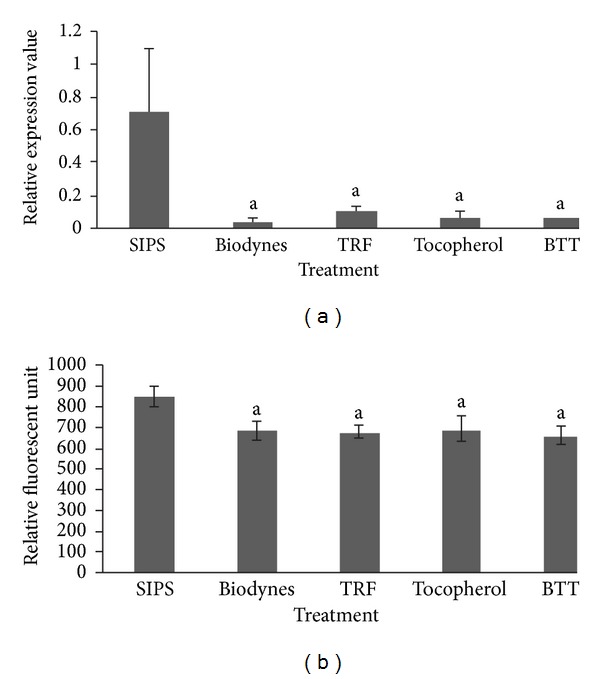
Effects of biodynes, TRF, and tocopherol on *MMP3 *gene expression and MMP-3 activity in HDFs. *MMP3 *expression (a). Biodynes, TRF, tocopherol, and BTT significantly downregulated *MMP3 *compared to SIPS. MMP-3 activity (b). Biodynes, TRF, tocopherol, and BTT significantly decreased MMP-3 activity. ^a^Denotes *P* < 0.05 compared to SIPS. Data are presented as the mean of three experiments ±S.D, *n* = 6.

**Figure 6:**
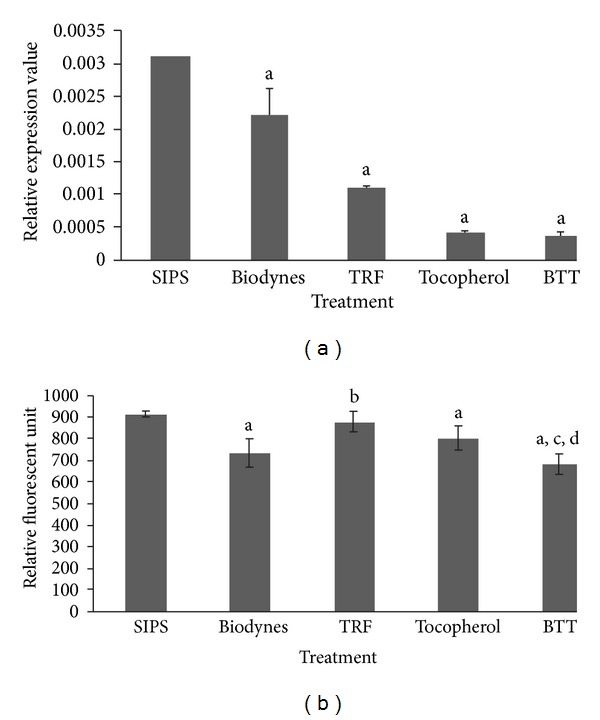
Effects of biodynes, TRF and tocopherol on *MMP9 *gene expression and MMP-9 activity in HDFs. *MMP9 *expression (a). Biodynes, TRF, tocopherol and BTT significantly down regulated *MMP9 *expression compared to SIPS. MMP-9 activity (b). Biodynes, tocopherol and BTT significantly decreased MMP-9 activity. ^a^Denotes *P* < 0.05 compared to SIPS, ^a^denotes *P* < 0.05 compared to biodynes, ^c^denotes *P* < 0.05 compared to TRF, ^d^denotes *P* < 0.05 compared to tocopherol. Data are presented as the mean of three experiments ±S.D, *n* = 6.

Similar findings were observed in the expression of *MMP3* gene and MMP-3 activity (Figures 5(a) and 5(b)), whereby biodynes, TRF, tocopherol, and BTT showed significant downregulation of *MMP3 *gene at 0.05-fold, 0.15-fold, 0.09-fold, and 0.08-fold, respectively (*P* < 0.05). Biodynes, TRF, tocopherol, and BTT also significantly downregulated *MMP9 *gene as compared to SIPS at 0.71-fold, 0.35-fold, 0.14-fold, and 0.12-fold, respectively (Figure 6(a)). Although biodynes, tocopherol, and BTT caused significant decrease in MMP-9 activity compared to SIPS, TRF-treated group showed higher MMP-9 activity as compared to biodynes (Figure 6(b)) (*P* < 0.05).

## 4. Discussion

This study evaluated the effects of biodynes, tocotrienol-rich fraction, and tocopherol in modulating collagen synthesis and degradation, in order to elucidate their underlying mechanism in preventing skin aging. HDFs were exposed to prolonged low dose of H_2_O_2_ to induce premature senescence. H_2_O_2_ has been used in various studies as the senescence induction agent that may produce similar characteristics to chronological aging on induced cells [15, 17, 21].

Progressive loss of skin tissue due to deterioration of cellular and extracellular matrix components of dermis is vital in skin aging. The dermis layer of the skin is mainly comprised of collagen fibers, consisting of several types of collagens such as types I, III, and V [5]. The turnover of collagen is crucially important for maintaining skin structure and function in which impaired production can lead to skin thinning and increase skin vulnerability. Thus, collagen synthesis and degradation were the focus of our study. Our results showed that exposure to prolonged low dose of hydrogen peroxide downregulated *COL1A1 *and *COL3A1 *genes with concomitant reduction in type I procollagen and type III procollagen synthesis. These findings are supported by report from previous studies which showed that type I collagen as the main component of dermis decreases during aging or photoaging [22, 23].

In addition to decreased collagen production, increased collagen degradation may result to collapsed fibroblasts during aging which contributed to the shift of aged skin appearance [13]. In this study, *MMP1*, *MMP2, MMP3, *and *MMP9 *genes were upregulated and MMP-1, MMP-2, and MMP-3 activities were increased in oxidative stress-induced fibroblasts. Oxidative stress caused by UV irradiation, ozone, H_2_O_2_, and free radicals may lead to activation of AP-1, accordingly increased MMPs expression and collagen degradation [7]. In addition, association of oxidative stress and the level of MMP-1 was reported by Fisher et al. [8] which is in line with our findings. Over expression of MMPs during aging observed in this study may results into collagen loss in the skin which has been reported to be prominent and was proposed as the hallmark to designate matrix-degrading phenotype in senescent cells [23–25].

In this study, we focused on the molecular mechanism of combined biodynes, TRF, and tocopherol in preventing skin aging. The findings from this study demonstrated that these compounds regulate collagen synthesis and degradation in cultured HDFs by upregulating collagen synthesis and downregulating MMPs expression at gene and protein levels. These effects could be attributed to the properties of these compounds. Biodynes is a collagen production promoting compound that enhances integrin synthesis in fibroblast cells and prevents or reduces wrinkle formation [26]. It also stimulates collagen synthesis by increasing oxygen uptake in human fibroblasts [19] and shows synergistic effect when combined with other active compounds which enhances products benefit [19].

Vitamin E, a potent antioxidant that scavenges ROS, has been used in various studies for the past few decades. In previous studies, vitamin E has been reported as an effective antiaging agent attributable to its antioxidant property. It exerted other beneficial effects such as in modulating signal transduction pathways [27–29]. Most of the studies used *α*-tocopherol as a representative of vitamin E [30, 31]. However, the lesser known form of vitamin E, tocotrienols, has been considered as greater options compared to tocopherols [32]. The tocotrienols have slightly higher antioxidant activity than the tocopherols, possess neuroprotective properties, and exhibit anti-cancer and cholesterol lowering properties that are often not exhibited by the tocopherols [13].

Our data showed that biodynes, TRF, and tocopherol upregulated collagen genes and increased the synthesis of procollagen. Interestingly, combined compounds gave a better effect in stimulating collagen synthesis as compared to treatment with single compound. Hence, combination of biodynes, TRF, and tocopherol is effective due to the contribution of each active compound. The effectiveness of vitamin E when employed in combination with biodynes can be postulated due to their antioxidant properties. However the molecular mechanism is not well understood, even though it may be related to the antioxidant properties of these active compounds and/or their effects in modulating genes expression and signaling pathways.

Several studies indicated that the MMPs are crucial for initiating the degradation of collagen. Collagenase is secreted into the extracellular spaces as a proenzyme and is later activated. It has been shown that MMP-2, stromelysin which is secreted together with collagenase from connective tissue cells in culture, is believed to play a role as an activator for collagenase. The presence of stromelysin is important for the expression of full collagenase activity [33]. Results obtained from this study showed that biodynes, TRF, and tocopherol decreased *MMPs* genes expression and reduced the activity of MMPs enzymes. Combined treatment with biodynes, TRF, and tocopherol exerted better effects in inhibiting collagen degradation as compared to single treatment. This effect may be explained by the report from recent findings on antiaging properties of tocotrienol [34]. Besides, biodynes have been reported to stimulate collagen synthesis by increasing oxygen uptake in human fibroblasts [19]. The findings from this study showed that biodynes, TRF, and tocopherol works synergistically and exerted better effect in modulating collagen synthesis and degradation.

## 5. Conclusion

Biodynes, TRF, and tocopherol effectively enhanced collagen synthesis and inhibited collagen degradation indicated by upregulation of collagen genes, type I and type III procollagen synthesis and down regulation of MMPs gene and reduced MMPs activity. These properties may indicate their potential in protecting the skin from aging.

## Figures and Tables

**Table 1:** Primers sequence for genes expression analysis.

Genes	Forward primer sequence	Reverse primer sequence
*GADPH *	CTT TGG TAT CGT GGA AGG ACT C	GTA GAG GCA GGG ATG ATG TTC T
*COL1A1 *	GTG CTA AAG GTG CCA ATG GT	ACC AGG TTC ACC GCT GTT AC
*COL3A1 *	CCA GGA GCT AAC GGT CTC AG	CAG GGT TTC CAT CTC TTC CA
*MMP1 *	ACA GCT TCC CAG CGA CTC TA	CAG GGT TTC AGC ATC TGG TT
*MMP2 *	AAC CCA GAT GTG GCC AAC TA	TGA TGT CTG CCT CTC CAT CA
*MMP3 *	GGC CAG GGA TTA ATG GAG AT	GGA ACC GAG TCA GGT CTG TG
*MMP9 *	CCA TTT CGA ACG ATG ACG AGT	CCT CGA AGA TGA AGG GGA AG
